# Investigation of infertility using endometrial organoids

**DOI:** 10.1530/REP-20-0428

**Published:** 2021-02-23

**Authors:** Konstantina Nikolakopoulou, Margherita Y Turco

**Affiliations:** 1 Department of Pathology, University of Cambridge, Cambridge, Cambridgeshire, UK; 2 Centre for Trophoblast Research, University of Cambridge, Cambridge, Cambridgeshire, UK

## Abstract

Infertility is a common problem in modern societies with significant socio-psychological implications for women. Therapeutic interventions are often needed which, depending on the cause, can either be medical treatment, surgical procedures or assisted reproductive technology (ART). However, the treatment of infertility is not always successful due to our limited understanding of the preparation of the lining of the uterus, the endometrium, for pregnancy. The endometrium is of central importance for successful reproduction as it is the site of placental implantation providing the interface between the mother and her baby. Due to the dynamic, structural and functional changes the endometrium undergoes throughout the menstrual cycle, it is challenging to study. A major advancement is the establishment of 3D organoid models of the human endometrium to study this dynamic tissue in health and disease. In this review, we describe the changes that the human endometrium undergoes through the different phases of the menstrual cycle in preparation for pregnancy. We discuss defects in the processes of endometrial repair, decidualization and acquisition of receptivity that are associated with infertility. Organoids could be utilized to investigate the underlying cellular and molecular mechanisms occurring in non-pregnant endometrium and early pregnancy. These studies may lead to therapeutic applications that could transform the treatment of reproductive failure.

## Introduction

The World Health Organization describes infertility as a ‘disease of the reproductive system defined by the failure to achieve a clinical pregnancy after 12 months or more of regular unprotected sexual intercourse’ (Zegers-Hochschild *et al.* 2017). It affects around 10% of couples of reproductive age and is a consequence of female or male reproductive dysfunction and/or low embryo quality (Vander Borght & Wyns 2018). In women, infertility is classified as primary or secondary, based on whether a clinical pregnancy has been previously achieved or not (Zegers-Hochschild *et al.* 2017). Despite the advances in assisted reproductive technology (ART), infertility is an increasing global public health issue.

Primary infertility of female origin is attributed to hormonal, functional or anatomical dysfunction of the organs of the reproductive tract. It is a complex disorder with a range of contributing factors including systemic diseases (diabetes and obesity), environmental (endocrine disruptors) and lifestyle (chronic stress and delayed childbearing) influences that can affect reproductive function (Vander Borght & Wyns 2018). Up to 30% of cases of infertility remain unexplained (Gelbaya *et al.* 2014). Known causes include ovarian dysfunction due to premature ovarian insufficiency, poor egg quality, polycystic ovary syndrome or fallopian tubal blockage, due to damage from infections by *Chlamydia trachomatis* or *Neisseria gonorrhoea*. Suboptimal function of the endometrium, the mucosal lining of the uterus, failing to achieve optimal decidualization and become receptive are a major cause of primary infertility (Cha *et al.* 2012).

The efficiency of reproduction is lower in humans compared to other mammals with only 30% of conceptions resulting in a live birth and the majority of these losses occurring preclinically (Macklon *et al.* 2002). In ART, methods for the selection of ‘good’ embryos prior to transfer include the assessment of embryo quality using a grading system that assesses the number of cells, fragmentation, speed and symmetry of cell division etc. The grading system changes in regard to the blastocyst stage. Yet, live birth rates after ART still remain low (Gleicher *et al.* 2019). Ovarian stimulation for fresh embryo transfer might impair endometrial receptivity and lead to asynchrony between the embryo and the endometrium in comparison to the transfer of frozen-thawed embryos in a natural cycle which results in higher pregnancy rates (Shapiro *et al.* 2013). Although later evidence shows that choosing to transfer fresh over frozen embryos and *vice versa* does not seem to significantly change the live birth rates (Wong *et al.* 2017), the matter is still under continuous investigation. Understanding how the normal endometrium is regulated to interact with the implanting blastocyst is essential to define the cellular and molecular mechanisms that are altered in a suboptimal endometrium.

The endometrium is a unique mucosa that consists of surface epithelium overlying glands with intervening stromal and immune cells. All these undergo profound phenotypic and transcriptional changes during the menstrual cycle in response to ovarian hormones (Garry *et al.* 2009, Munro *et al.* 2010, Ruiz-Alonso *et al.* 2012). Current knowledge of human endometrial function is based on decades of studies using endometrial tissues and a range of *in vivo* and *in vitro* models. Animal models, including non-human primates (monkeys, baboons) (Stouffer & Woodruff 2017) and rodents, have provided important insights (Fullerton *et al.* 2017). However, animal studies are labour and cost-intensive and not all aspects of human endometrium are recapitulated. *In vitro* models offer a more reproducible alternative. Immortalized human endometrial stromal cells have been used as reporter lines to study genes involved in decidualization (Haller *et al.* 2019). Commonly used cell lines derived from endometrial adenocarcinomas (Ishikawa cells and ECC1) are useful to test the effects of oestrogen and progesterone as well as to model the luminal epithelium to study implantation *in vitro* (Mo *et al.* 2006, Zhang *et al.* 2012). These systems suffer from a failure to fully reflect the normal physiological characteristics of the endometrial epithelium. In addition, their 2D nature prevents the cell–cell interactions which are characteristic of the complex microenvironment *in vivo*. The establishment of tissue-derived 3D culture systems or organoids of the female reproductive tract and the placenta offer powerful models for the study of these tissues and pregnancy *in vitro* (Alzamil *et al.* 2020, Heidari-Khoei *et al.* 2020, Ojosnegros *et al.* 2021).

The physiology of the endometrium is a determining factor in the establishment and maintenance of pregnancy as it provides the site of implantation and early nutrition to the conceptus during the first few weeks of pregnancy (Burton *et al.* 2002). In this review, we describe the functional and morphological changes of the endometrium during the menstrual cycle and in pregnancy. We discuss how both the endometrial physiology and the defects that might arise in distinct temporal stages can be studied using organoids. Understanding the pathophysiology of infertility due to uterine factors using organoid technology will help pave the way for the development of strategies to diagnose reproductive failure and to design personalized therapeutic interventions.

## Endometrial organoid culture systems

**Figure 1:**
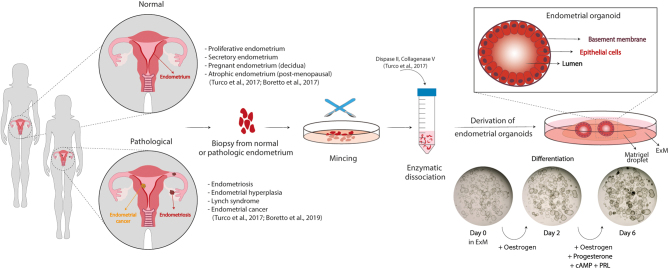
Organoids of the human endometrium. Organoids can be derived from tissue biopsies from non-pregnant women in the proliferative or secretory phase of the menstrual cycle or from pregnant and postmenopausal women. The sample is minced mechanically and enzymatically to obtain epithelial isolates which are resuspended in an extracellular matrix (Matrigel) to allow self-organization of the organoids into a cystic structure formed by columnar epithelial cells lining a central lumen. They can be propagated long-term in a chemically defined medium (Expansion medium, ExM) containing nicotinamide, supplements (N2, B27), activators of WNT signalling (RSPO1 and CHIR99021), growth factors (EGF, HGF, and FGF10) and inhibitors of TGFB and BMP signalling pathways (A83-01 and Noggin, respectively). They recapitulate the morphological, molecular and functional features of endometrial glands *in vivo* and respond to steroid hormones, oestrogen and progesterone. To induce differentiation, they are primed with oestrogen for 2 days and then treated with a combination of oestrogen, progesterone, prolactin and cAMP for another 4 days. Brightfield images depict morphological changes in an organoid culture before (day 0), during (day 2) and after (day 6) their differentiation with hormones. Organoids with thicker epithelia and secretions in the lumen are visible after full hormonal stimulation. Organoids can also be derived from pathological endometrium including endometriosis, endometrial hyperplasia and cancer.

Endometrial organoids are 3D cultures of the epithelium. They can be derived from endometrial tissue from all stages of the menstrual cycle, as well as from decidua and atrophic endometrium (Fig. 1). Isolated gland fragments are embedded into an extracellular matrix (typically the commercially available Matrigel) and are grown in a chemically-defined medium designed to sustain WNT and MAPK signalling whilst inhibiting TGFB and BMP pathways (Boretto *et al.* 2017, Turco *et al.* 2017). The organoids are spheroidal structures, around several hundred microns in size, with a central lumen that is lined by a single columnar epithelium with microvilli on their apical surface (Fig. 1). They can be expanded long-term (>6 months) without genetic alterations. The organoids respond functionally to ovarian hormones and placental factors, demonstrated by their expression of oestrogen and progesterone receptors and production of uterine glandular products (Turco *et al.* 2017). The ability of the organoids to recapitulate the changes of the endometrial epithelium across the menstrual cycle has also been demonstrated by comparing the hormonally stimulated organoids to endometrial tissue by single-cell RNA sequencing (Garcia-Alonso *et al.* 2021). Organoids can also be derived from pathological endometrial tissues including endometriosis and endometrial carcinomas (Boretto *et al.* 2019). Collectively, these *in vitro* models allow the study of endometrial physiology and disease in a reproducible and standardized manner and are a promising tool for the investigation of infertility.

## Organoids for the study of the normal endometrium

### Menstruation, tissue repair and regeneration

**Figure 2:**
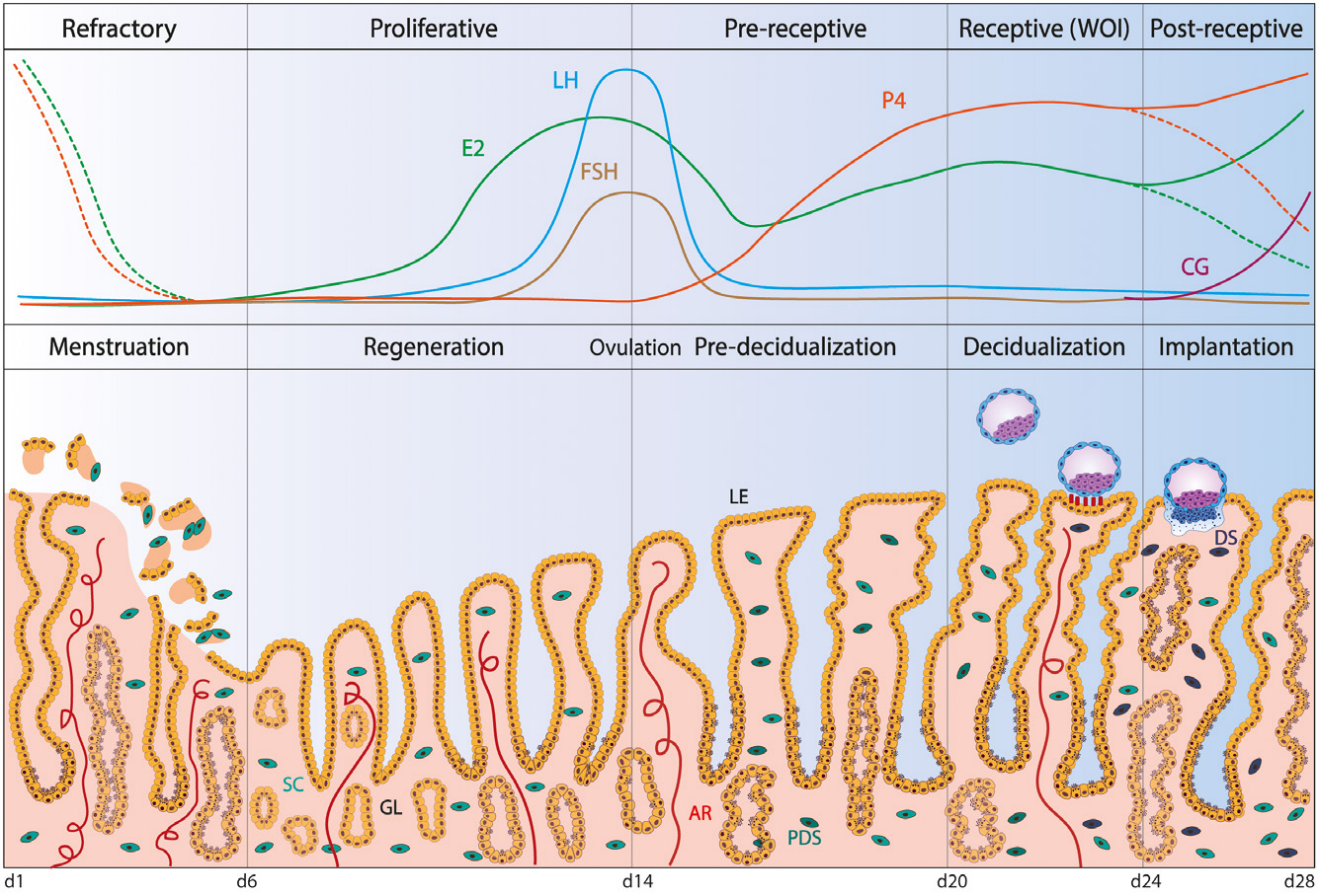
Endometrial changes across the menstrual cycle. The endometrium undergoes significant changes during the course of the menstrual cycle which in humans lasts approximately 28 days. Menstruation is the first phase of the cycle, during which the spiral arteries (AR) contract, resulting in ischaemia and shedding of the functional layer of the tissue, including both epithelial and stromal compartments. Repair starts almost simultaneously. In the proliferative phase, regeneration is driven by rising levels of oestrogen (E_2_) from the ovarian follicle that stimulate proliferation of epithelial, stromal and endothelial cells. Numerous developing glands of tortuous morphology are characteristic of the proliferative phase. At midcycle, the surge of gonadotropins, follicle-stimulating hormone (FSH) and luteinizing hormone (LH) released from the anterior pituitary induce ovulation. The release of the oocyte marks the beginning of the secretory phase during which the corpus luteum forms from the ruptured ovarian follicle and secretes progesterone (P_4_), the hormone responsible for preparation of the uterine environment for pregnancy. During this phase, the endometrial glands (GL) become cork-screw shaped and twisted whilst their secretory potential maximizes. The stromal cells (SC) undergo concomitant changes differentiating intopre-decidualized stromal first (PDS) and then decidualized stromal (DS) cells. Rising levels of E_2_ in combination with P_4_ mark the window of implantation (WOI), a limited time frame during which the implantation happens. The blastocyst attaches to the endometrium through projections (pinopodes) that form at the apical side of the luminal epithelium (LE). In the absence of a blastocyst, the WOI spontaneously becomes refractory due to the rapid decrease in P_4_ levels leading to menstruation, thus resetting the cycle. Conversely, an implanting blastocyst secretes chorionic gonadotropin (CG) to maintain the secretion of P_4_ from the corpus luteum, thereby supporting pregnancy.

During the menstrual cycle, which in humans last 26–32 days, the endometrium undergoes shedding, repair, regeneration and remodelling to provide a ‘fertile soil’ for implantation of the embryo (Evans *et al.* 2016). Because of this extraordinary plasticity, it has been challenging to study as access to samples throughout the cycle is needed, together with models that can recapitulate this process. Menstruation, the shedding of the functional layer of the endometrium (days 1–5), occurs in the absence of conception and embryonic implantation. The trigger for menstruation is the regression of the corpus luteum leading to a reduction in levels of ovarian hormones, oestrogen and progesterone (Fig. 2). Vasoconstriction of uterine blood vessels and breakdown of the stratum functionalis then follow (Maybin & Critchley 2015). To ensure tissue integrity, endometrial repair starts almost immediately after the onset of menstruation (days 2–5) along with re-epithelization and revascularization (Fig. 2) (Ludwig & Spornitz 1991). Although oestrogen is not essential for endometrial repair, it is required for efficient regeneration during the proliferative or follicular phase of the menstrual cycle (days 6–13) (Fig. 2). Oestrogen receptor (*ESR1*) expression is highest during this phase in both the epithelial and stromal compartments where maximal levels of mitoses are seen (Lessey *et al.* 1988).

Our understanding of the mechanisms that regulate the breakdown and regeneration of the endometrium each month is surprisingly limited. During the first half of the cycle, epithelial and stromal cells proliferate. Although there is a consensus about stem/progenitor cells with clonogenic properties being responsible for endometrial regeneration, their exact identity and location have been controversial. Putative progenitors include epithelial cells in basal glands (SSEA-1^+^, SOX9^+^, CDH2), epithelial cells at the surface epithelium (*LGR5*^+^), stem cells likely to be pericytes (SUSD2^+^, CD146^+^CD140B), stromal fibroblasts from menstrual blood, endometrial and decidual side-population cells (CD31^+^ endothelial cells and CD140B CD146^+^ pericytes) and bone marrow-derived cells (Gargett *et al.* 2016, Nguyen *et al.* 2017, Tempest *et al.* 2018, Hapangama *et al.* 2019). Thus, this central question of which cells drive the regenerative process remains unanswered.

**Figure 3:**
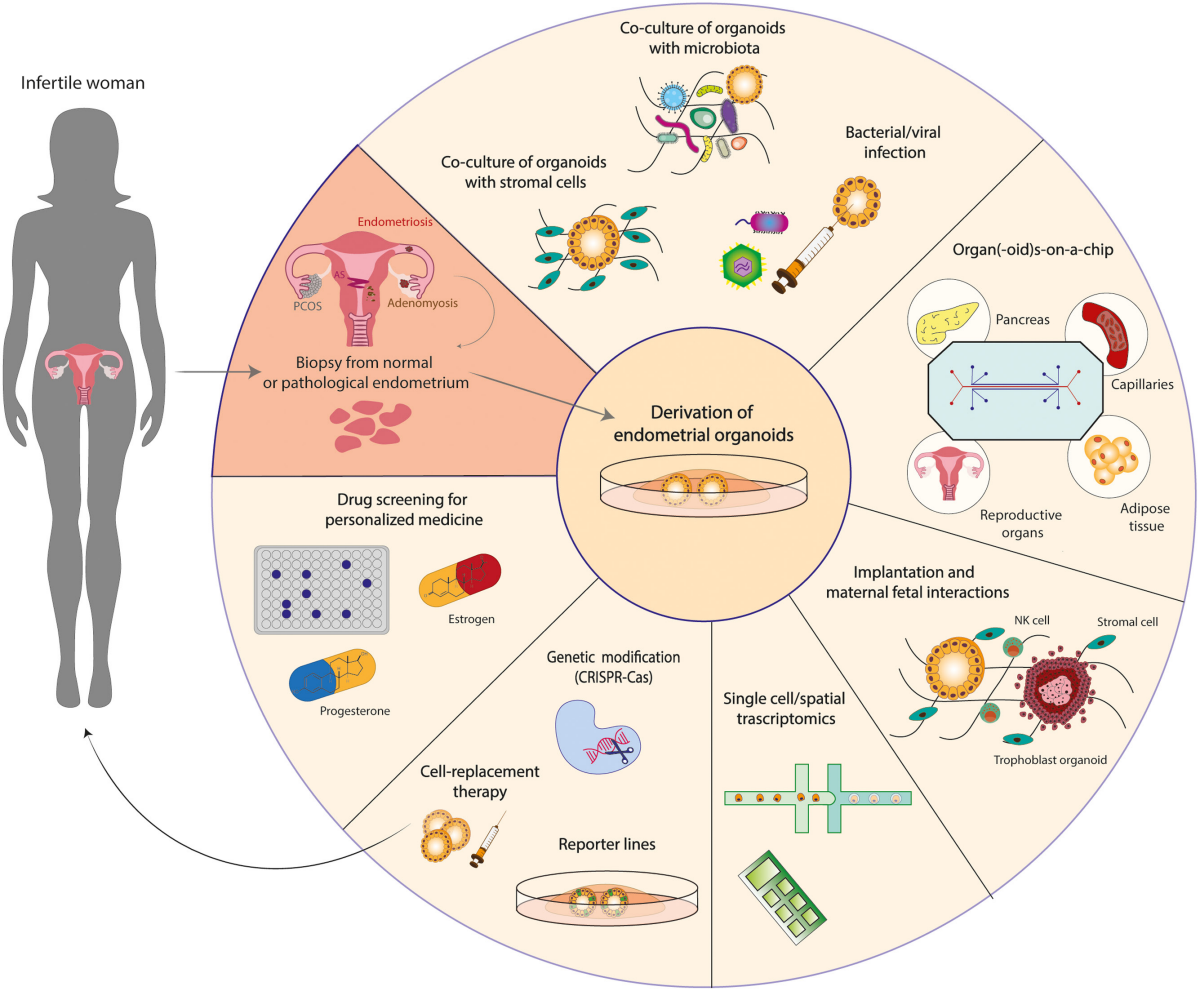
Applications of endometrial organoids in infertility. Organoids can be derived from biopsies of normal or pathological endometrium (endometriosis or carcinoma). Organoids from women suffering from other uterine disorders such as Asherman’s syndrome (AS), adenomyosis or other disorders that affect the endometrium indirectly, like polycystic ovary syndrome (PCOS), are yet to be established. The organoids consist of epithelial cells but isolation of stromal cells from the same biopsies will allow the creation of complex co-culture systems that combine both cell types facilitating the study of decidualization. Exposure of the organoids to commensal or pathogenic microorganisms (viruses, bacteria) will reveal how infection affects epithelial integrity and fertility. Implementation of bioengineering technologies for the combination of organoids from reproductive organs (ovaries, endometrium) and other organs/tissues- (pancreas, adipose tissue, capillaries) on-a-chip will lead to the creation of more dynamic *in vitro* models. Endometrial organoids could be co-cultured with trophoblast organoids derived from the first-trimester placentas, to study the signals that mediate the interaction between the fetus and the mother. Addition of uterine natural killer (NK) cells will help elucidate how maternal immune cells affect trophoblast proliferation and differentiation and shed light into obstetric disorders that arise due to placentation failure. Another application of endometrial organoids is their use in single cell and spatial transcriptomic technologies for the identification and localization of progenitor cells. Potentially identified markers of progenitor cells could then be used to genetically edit organoids to create reporter lines and monitor their expression in different conditions. In the future, organoids could provide an alternative therapy for the replacement of scarred endometrium in women with AS. Women suffering from endometriosis could also benefit from replacement of the progesterone-resistant endometrium with organoids, genetically manipulated to express progesterone receptors and thus respond to progesterone. Translational applications of the endometrial organoids include drug testing; testing the dosage and response to oestrogen and progesterone to offer tailored hormonal treatment for women prior to *in vitro* fertilization (IVF).

Endometrial organoids proliferate in response to signalling cues provided by the culture medium and differentiate upon treatment with hormones (Fig. 1). Their clonal ability and potential to grow long-term make them a unique tool to address the identity of epithelial progenitors and to mimic/reproduce changes during the menstrual cycle *in vitro*. Whether it is the withdrawal of the hormones from the culture medium or/and mechanical disruption (passaging) that facilitates their subsequent regrowth has not been addressed. Transcriptomic approaches allow the generation of reference datasets to identify the different epithelial populations *in vivo* and infer their potential roles in the function of the endometrium. For example, single-cell RNA sequencing analysis of organoids has identified a putative stem cell population that decreased when they were treated with oestrogen, progesterone and cyclic AMP (cAMP) (Fitzgerald *et al.* 2019). The dynamics of *SOX9* and *LGR5* expression in the human uterus during the menstrual cycle was refined by the generation of a reference map using single-cell and spatial transcriptomics (Garcia-Alonso *et al.* 2021). This has revealed that *SOX9*^+^ cells segregate into a non-cycling *LGR5*^+^ population at the luminal epithelium and a *LGR5*^−^ population in the deeper glands. To investigate whether these markers are specific for cells with stem cell/progenitor potential, reporter organoid lines for these genes could be generated using gene editing approaches. For instance, genes of interest linked to a fluorescent tag could be introduced (knocked-in) into single cells using the CRISPR/Cas9 technology (Artegiani *et al.* 2020). These genetically modified organoid lines would allow the investigation of pathways regulating proliferation/regeneration (Fig. 3). Such studies will help identify progenitors of the endometrial epithelium and how their altered behaviour may be involved in disorders, such as endometriosis affecting fertility. For example, it has been shown that in women with endometriosis, there is an increase in the number of SOX9^+^ SSEA1^+^ cells in the functional layer that can generate ectopic endometriotic lesions in 3D culture (Hapangama *et al.* 2019).

### Pre-receptive and receptive phases

Upon pregnancy, the endometrium continues its differentiation and transforms into decidua. Decidualization in humans (and other species that menstruate) occurs spontaneously, unlike in mice. Following ovulation (day 14), the endometrium undergoes substantial remodelling of all cellular elements to become receptive for implantation in the mid-secretory or luteal phase (Fig. 2). Initially, increasing levels of progesterone prime the endometrium to enter a pre-receptive state (days 15–19). Although the luminal epithelium is still refractive to implantation, the glandular epithelium becomes increasingly columnar with the appearance of characteristic subnuclear vacuoles. As decidualization proceeds, the uterine glands increase their production of ‘uterine milk’ containing components like osteopontin, glycodelin and EGF (Burton *et al.* 2020). The stromal cells encircling spiral arteries enlarge to acquire a secretory phenotype and produce basement membrane components. As the secretory phase progresses, there is an increased production of prolactin (PRL) and insulin-like growth factor-binding protein 1 (IGFBP1) (Massimiani *et al.* 2019). In order to facilitate implantation, the luminal epithelium also undergoes changes to become adhesive. Pinopodes appear along with the expression of specific integrins, glycoproteins and cadherins (Creus *et al.* 2002).

The window of implantation (WOI) (days 20–24) marks the phase during which the endometrium is receptive and the implantation of the blastocyst can occur (Fig. 2). Although there are several assays for receptivity (e.g. endometrial thickness, volume, pattern, blood flow), their accuracy is questionable and, thus, their clinical use is poor (Craciunas *et al.* 2019). On the contrary, genetic signatures and molecular biomarkers of receptivity are more promising. An endometrial receptivity array can be used to classify endometrial biopsies according to their transcriptomic profile, based on which women can then undergo personalized embryo transfer (Díaz-Gimeno *et al.* 2011). This allows the proper timing of the transfer according to the receptive status of the endometrium and evidence shows that it can efficiently predict pregnancy rates (Díaz-Gimeno *et al.* 2017). Now, the transcriptomic transition of the endometrium across stages of the menstrual cycle has been refined accounting for the different cell types using next-generation sequencing technologies (Wang *et al.* 2020). Abrupt and discontinuous transcriptional activation of the endometrial epithelium followed by a continuous transition of the fibroblasts could be used to predict the start of the WOI more accurately (Wang *et al.* 2020).

**Figure 4:**
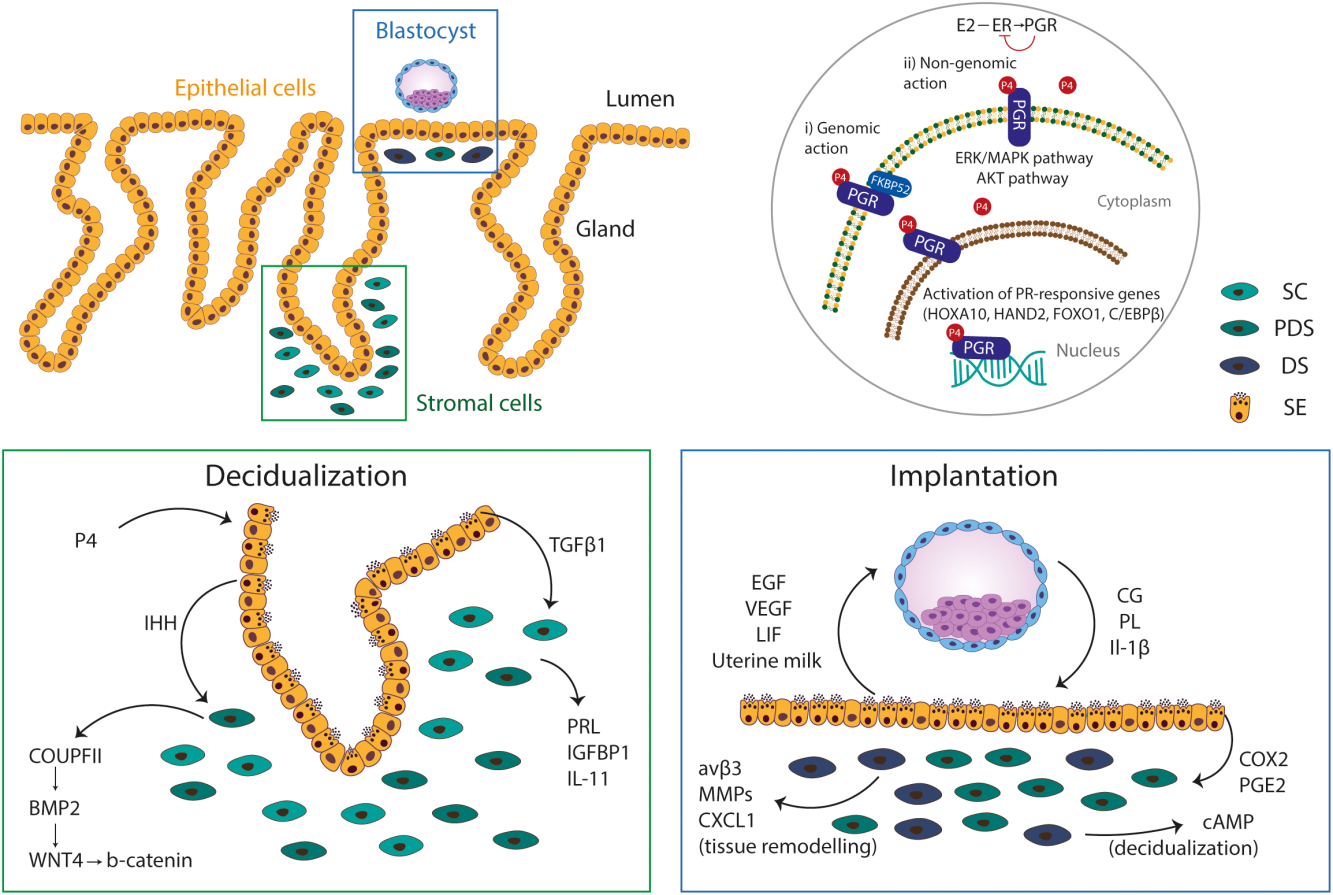
Signalling pathways crucial for decidualization and implantation. Decidualization and implantation rely on continuous cellular interactions between epithelial cells at the surface and in glands, stromal cells and the incoming blastocyst (top left panel). Progesterone (PG) mediates its function through progesterone receptor (PGR) whose expression is induced by the action of oestrogen (E_2_) through oestrogen receptor (ESR1), and in turn, PGR inhibits ESR to create a negative feedback loop system. Upon binding of P_4_, PGR acts as a transcription factor which translocates to the nucleus to mediate genomic actions. Homeobox A10 (HOXA10), Forkhead Box O1 (FOXO1), heart and neural crest derivatives expressed 2 (HAND2) and CCAAT/Enhancer Binding Protein-β (C/EBPβ) are transcription factors that are regulated by progesterone and are indispensable for decidualization. FK506 binding protein prolyl isomerase 4 (FKBP52) is a PGR chaperon that promotes the activity of the receptor during decidualization. PGR at the surface of the cells can also trigger downstream molecules to activate the extracellular signal-regulated kinase/mitogen-activated protein kinase (ERK/MAPK) and Protein Kinase B (AKT) pathways and, thus, mediate non-genomic actions (top right panel). Progesterone activates the expression of Indian hedgehog (IHH) in the epithelium, a morphogen that promotes the decidualization of stromal cells via activation of chicken ovalbumin upstream promoter-transcription factor II (COUPTFII) that subsequently regulates the expression of bone morphogenetic protein 2 (BMP2). BMP2 is a morphogen of the TGFB pathway, acting through Wingless 4 (WNT4). TGFB1 is another member of the TGFβ pathway secreted by epithelial cells. Subsequently, stromal cells are stimulated to produce prolactin (PRL), insulin-like growth factor-binding protein 1 (IGFBP1) and interleukin 11 (IL-11), classic markers of decidualization (bottom left panel). Upon attachment to the uterus, trophoblast secretes hormones, chorionic gonadotropin (CG) and placental lactogen (PL), as well as cytokines like interleukin 1β (IL1B). In turn, epithelial glands secrete uterine milk, a cocktail of glycoproteins (glycodelin-A, osteopontin, uteroglobin), glycogen, lipids, as well as, growth factors (leukaemia inhibitory factor (LIF), epidermal growth factor (EGF), vascular endothelial growth factor (VEGF). In addition, IL1B and CG reinforce decidualization by promoting cyclooxygenase-2 (COX2) and prostaglandin E synthase (PGE2) expression by epithelial cells and subsequently augmenting cAMP production from stromal cells. IL1β also acts on maternal stromal cells to promote expression of αvβ3 integrin, matrix metalloproteinases (MMPs) and C-X-C motif chemokine 10 (CXCL10) which are necessary for tissue remodelling and angiogenesis to support trophoblast invasion (bottom right panel). SC, stromal cell; PDS, pre-decidualized stromal cell; DS, decidualized stromal cell; SE, secretory epithelial cell.

Decidualization is mediated by hormonal signalling through oestrogen and progesterone. Oestrogen acts through the oestrogen receptor α (ESR1) to enhance the expression of progesterone receptor (PGR) allowing the endometrium to respond to the elevated levels of progesterone during the secretory phase (Massimiani *et al.* 2019) (Fig. 4). PGR is expressed by both epithelial and stromal cells (Snijders *et al.* 1992) and is activated by progesterone to initiate decidualization through epithelial–stromal cell interactions. Progesterone-regulated transcription factors (CCAAT/Enhancer Binding Protein-β and the Homeobox family proteins A10) and progesterone mediators (Bone Morphogenetic Protein-2, Indian Hedgehog, Forkhead Box O1, Heart and Neural Crest Derivatives expressed 2) are crucial for decidualization (Massimiani *et al.* 2019) (Fig. 4).

Endometrial organoids can be used to study decidualization. When stimulated with oestrogen followed by progesterone, they upregulate ESR1 and PGR and secrete uterine milk products including glycodelin and osteopontin accompanied by transcriptomic changes with downregulation of putative stem cell markers found in other epithelia (*SOX9*, *PROM1*, *AXIN2* and *LRIG1*) (Turco *et al.* 2017). This will facilitate the study of epithelial-specific responses to ovarian hormones and, together with CRISPR/Cas gene-editing technologies, the signalling cascades could then be examined in detail. For example, oestrogen and progesterone receptors could be knocked-out either separately or in combination in single cells from established endometrial organoids. These would then be clonally expanded into knock-out lines to assess the exact role of hormone signalling in differentiation and acquisition of a secretory glandular profile. CRISPR/Cas gene targeting has been successfully achieved in different organoid systems (Hendriks *et al.* 2020). Though similar approaches have been employed for targeting endometrial cell lines (Kinnear *et al.* 2019, Wang *et al.* 2019), this has not yet been reported for endometrial organoids. Technical challenges include the requirement of high cell numbers and optimal method of delivery of targeting reagents. Using these approaches, important questions like how progesterone resistance in patients with endometriosis could negatively impact decidualization and successful preparation for pregnancy could be addressed. There are also new methods to co-culture endometrial organoids with stromal cells in a collagen scaffold providing opportunities for *in vitro* modelling of the endometrium to study stromal–epithelial interactions driving decidualization (Abbas *et al.* 2020) (Fig. 3).

### Implantation and post-receptive phase

In the absence of a viable embryo, the WOI closes and the endometrium transits into a non-receptive phase (days 24–28). The corpus luteum involutes with decreasing progesterone levels resulting in menstruation (Fig. 2). The release of chorionic gonadotropin from a blastocyst during the WOI, on the other hand, aids the maintenance of the corpus luteum and decidualization continues (Fig. 2). The trophectoderm surrounding the inner cell mass makes contact with the luminal surface of the receptive endometrium through a finely regulated process of apposition, attachment and implantation. Attachment of the blastocyst reinforces decidualization of the stromal compartment through the secretion of signalling molecules and, in response, the latter signals back to support blastocyst survival (Fig. 4). These interactions are fundamental for the proper establishment of the maternal–fetal interface. However, the characteristics of a receptive decidualized endometrium and the nature of the signals between decidua and embryo that support pregnancy remain elusive.

In humans, implantation is particularly intrusive and the trophectoderm rapidly invades the endometrial stroma once it has breached the luminal epithelium. In comparison to other species, the human blastocyst burrows in the surface epithelium, which grows over it and surrounds it. Specialized placental cells, extravillous trophoblast, invade the uterine spiral arteries in order to establish the blood supply for the fetal–placental unit (Turco & Moffett 2019). A balanced invasion of the trophoblast into the decidua is essential for a successful pregnancy; too shallow invasion results in insufficient spiral artery remodelling resulting in pre-eclampsia, miscarriage or still-birth whilst too deep invasion results in the accreta spectrum (Jauniaux *et al.* 2018). The maternal–fetal interactions can now be investigated using human trophoblast organoids from the early human placenta (Haider *et al.* 2018, Turco *et al.* 2018) that retain *in vivo* features of early trophoblast and secrete placental-specific peptides and hormones, including chorionic gonadotropin (Turco *et al.* 2018). Trophoblast organoids are a powerful tool for studying the development of the human placenta and its interactions with the decidua as they can also differentiate into invading extravillous trophoblast cells (Turco *et al.* 2018).

Uterine natural killer (uNK) cells make up ~70% of CD45^+^ leukocytes cells within the decidua (King *et al.* 1989, Vento-Tormo *et al.* 2018). uNK cells proliferate *in situ* in the secretory phase under the influence of progesterone when other cellular elements are differentiating and during pregnancy, they are found at the site of placentation close to infiltrating trophoblast cells and around the maternal spiral arteries (Moffett & Shreeve 2015). The exact functions of uNK cells are still unknown but genetic studies of pre-eclampsia suggest that they are likely to regulate the invasion of trophoblast cells maintaining the balance between excessive and deficient trophoblast intrusion (Moffett & Colucci 2014). This fundamental question can be addressed by co-culturing uNK cells with trophoblast organoids to identify factors secreted by uNK cells affecting the growth, differentiation and potential for invasion of trophoblast (Fig. 3). uNK cells are never in contact with the inner cell mass or developing embryo and cannot cause ‘fetal rejection’. There is also no evidence that uNK cells are responsible for reproductive failure in women with failed IVF and treatments aiming to ‘suppress’ uNK cells are not without risk to healthy women (Moffett & Shreeve 2015). Current evidence indicates that recognition of fetal trophoblast cells activates the maternal NK cells, through certain ligand-receptor combinations, which could then enhance placentation (Xiong *et al.* 2013).

Besides organoids, culture methods for human embryos, together with transcriptomic and bioengineering technologies, provide additional experimental approaches for the study of early human development (Deglincerti *et al.* 2016, Shahbazi *et al.* 2016, Cermola *et al.* 2021). Stem cell-derived pre-implantation embryos ‘blastoids’ could provide a solution for overcoming the ethical difficulties of using human embryos (Rivron *et al.* 2018, Zheng *et al.* 2019). Endometrial models could then be co-cultured with embryos/blastoids and trophoblast organoids to create an implantation model (Ban *et al.* 2020) (Fig. 3). Although there are many challenges in combining different cell types, such a model would also offer a unique opportunity to explore the secretory profile of the endometrium during pregnancy and its effect on the trophoblast. The aim is to identify markers of endometrial receptivity or critical regulators of the maternal–fetal cross-talk for the development of predictive tests and therapeutic interventions for tackling compromised fertility related to poor endometrial receptivity.

## Investigation of abnormal endometrial function and infertility using organoids

### Defective endometrial regeneration and proliferation

Altered function or regulation of endometrial progenitors and their niche are likely to be responsible for some endometrial disorders. Dysregulated proliferation is seen in endometriosis, hyperplasia and carcinomas or during the insufficient growth of the endometrium (Gargett *et al.* 2016). Although controversy exists on the clinical significance of reduced thickness in women undergoing ART, a thin endometrium unresponsive to oestrogen stimulation has been associated with failure of implantation. A recent retrospective study shows that decreased endometrial thickness (<7 mm) on the day of ovulation, but not on the day of embryo transfer, is associated with low pregnancy rates (Nishihara *et al.* 2020). Even in women without ovarian stimulation, a thin endometrium may be considered a negative prognostic factor for pregnancy (von Wolff *et al.* 2018). Several factors could be responsible for a thin endometrium: low oestrogen levels and inadequate blood flow due to lifestyle factors (increased BMI, birth control pills, ovulation stimulating drugs).

Canonical Wnt/β-catenin signalling is essential for the development of the human uterus (Habiba *et al.* 2021). The WNT pathway also plays a role in adulthood as it regulates endometrial cell proliferation and homeostasis after menstruation. Perturbations in WNT signalling have been associated with infertility (Sonderegger *et al.* 2010). Enhancement of the activity of WNT pathway by oestrogen and inhibition by progesterone fine tune its action and prevent neoplastic transformation (Wang *et al.* 2010). This effect of WNT signalling is seen in organoids as the withdrawal of R-spondin, a growth factor that enhances this pathway, from the culture medium causes a reduction in size and number of organoids (Boretto *et al.* 2017, Turco *et al.* 2017). WNT ligands (*WNT3A*, *WNT4* etc.) but not R-spondins are expressed by organoids explaining the requirement for external addition of R-spondin but not WNT3A (Boretto *et al.* 2017). Components of the WNT pathway, including its target genes, have been linked to endometriosis and endometrial cancer (Boretto *et al.* 2019). The growth of organoids from ectopic endometriotic tissue is enhanced by R-spondin but reduced when the WNT pathway is inhibited. Transcriptomic analysis of ectopic organoids further highlights the expression of WNT pathway genes (*CTNNA2*, *LEF1*, *WNT11*). Organoids from endometrial low-grade tumours also highly express WNT target genes (*AXIN2*, *LEF1*, *CMYC*, *TCF4*, *LGR6*) (Boretto *et al.* 2019). Furthermore, integration of scRNAseq data of the human uterus with genome-wide association studies (GWAS) shows endometrial fibroblasts expressing WNT ligands involved in endometriosis (*WNT4*, *RSPO3*) and endometrial cancer (*RSPO1*) which could bind WNT receptors in epithelial cells (Garcia-Alonso *et al.* 2021). Because of its possible implication in altered proliferative and regenerative capacity in i women with unexplained infertility, Wnt signalling could be studied in organoids derived from these women. Another interesting application would be to prolong exposure of the organoids to oestrogen and investigate changes in WNT components and test whether a hyperplastic profile emerges.

### Disorders affecting endometrial function and decidualization

Defective decidualization impedes the ability of the endometrium to receive and support the survival of the embryo. Apart from infertility, it has been implicated in pregnancy complications including pre-eclampsia, fetal growth restriction and preterm birth (Cha *et al.* 2012, Conrad *et al.* 2017). Decidualization defects are also seen in disorders that lead to hormonal and metabolic dysfunction of the endometrium, including endometriosis, adenomyosis, and polycystic ovary syndrome (PCOS). Endometriosis is a complex disease affecting ~10% of women of reproductive age, characterized by endometrial deposits outside the uterine cavity, including pelvic peritoneal surfaces, ovaries, ligaments, bladder or the bowel (Holoch & Lessey 2010). Painful periods and intercourse, chronic pelvic pain and abnormal bleeding are the most common symptoms. Around 50% of women with endometriosis, in particular those <35 years, have fertility issues (Holoch & Lessey 2010). GWAS meta-analysis studies identified susceptible genes for endometriosis including those involved in oestrogen-regulated pathways (*OSR1*, *GREB1*) (Sapkota *et al.* 2017). This reinforces the importance of oestrogen dominance in endometriosis and, hence, increased proliferation (Marquardt *et al.* 2019). Progesterone resistance, the inability to respond to progesterone, has been commonly described in both ectopic and eutopic endometrium in endometriosis, attributed to dysfunction of the PGR itself or its downstream mediators (Marquardt *et al.* 2019). Failure to respond properly to progesterone not only results in defective decidualization but also in growth of lesions due to a failure to counteract oestrogen-induced proliferation (Marquardt *et al.* 2019). A recent study has shed further mechanistic insight that may question progesterone ‘resistance’ (Houshdaran *et al.* 2020). Although endometrial stromal fibroblasts from women with endometriosis do not fully decidualize, they respond to progesterone differently from normal cells (Houshdaran *et al.* 2020).

Altered progesterone response is not restricted to endometriosis and may play a role in other endometrial disorders. Adenomyosis, characterized by the presence of endometrial glands within the myometrium, is a poorly understood disease. It is commonly linked to endometriosis with the key characteristic being hyperestrogenism, which may result in progesterone resistance in the eutopic endometrium (Benagiano *et al.* 2014). Polycystic ovary syndrome (PCOS) could also affect the endometrium indirectly. Anovulatory cycles are a major feature of PCOS meaning that the corpus luteum does not form and progesterone is absent. The expression of PGR is altered and genetic analyses revealed impaired response to progesterone in women with PCOS. However, the exact molecular mechanism underlying progesterone resistance in PCOS is not understood (Li *et al.* 2014). Hyperandrogenism is likely another feature of PCOS contributing to impaired endometrial function (Simitsidellis *et al.* 2018). Elevated androgens result in delayed decidualization of stromal cells which produce limited amounts of prolactin *in vitro* (Younas *et al.* 2019). As a consequence, the endometrium would fail to acquire a receptive state in time to support implantation. Understanding the relationship between altered hormonal responses and impaired decidualization is crucial for assessing the receptive capacity of an abnormal endometrium.

Organoids have been generated from eutopic and ectopic endometrium of women with endometriosis. They recapitulate the morphological and molecular characteristics of the native endometriotic lesions and reproduce the disease when injected into the peritoneal cavity of mice. In addition, glycodelin (*PAEP*), a progesterone-regulated gene, was downregulated in the organoids at different stages of endometriosis. On the contrary, *SOX9* was upregulated further supporting the hypothesis that an altered progesterone response and enhanced proliferation is driving endometriosis (Boretto *et al.* 2019). In future, establishment of endometrial organoids from patients with adenomyosis or PCOS will allow further investigation on how receptivity is regulated (Fig. 3) and lead to appropriate therapies to enhance fertility in women with defective endometrium.

An interesting perspective has highlighted variable methylation patterns of the Human Homeobox (HOX) genes and their cofactors between eutopic and ectopic organoids and tissues from women with endometriosis (Esfandiari *et al.* 2021). These epigenetic changes could be used as a prognostic factor for endometriosis. More specifically, organoids derived from girls likely to inherit a harmful epigenetic trait from their mothers suffering from endometriosis could be used to predict their chances of developing endometriosis (Koninckx *et al.* 2021).

### Lifestyle and environmental factors affecting endometrial function

Delayed maternal age and high BMI are associated with sub/infertility (Vander Borght & Wyns 2018). Spontaneous abortions in the first trimester of pregnancy are common in older women due to chromosome mis-segregations in the oocyte leading to karyotypic imbalances in the offspring. In the absence of any karyotypic abnormality, advanced maternal age still contributes to pregnancy complications, namely fetal growth restriction, pre-eclampsia and stillbirth due to disordered embryo–maternal interactions. Aged mice have a reduced number of NK and dendritic cells which could have a negative effect on placentation, whilst stromal cells present a blunted response to decidualization signals (Woods *et al.* 2017). Apart from poor oocytes, the mechanisms by which increased maternal age impede endometrial function and normal pregnancy are unclear. The influence of obesity, along with other lifestyle factors (smoking, lack of exercise, stress, drugs, environmental pollutants), on fertility is an important current discussion (Silvestris *et al.* 2018). Obese mice have smaller implantation sites and deciduomas, whilst* in vitro* human endometrial stromal cells cultured in the presence of fatty acids demonstrate lower expression of decidualization markers (PRL, IGFBP1) (Rhee *et al.* 2016). Given that many aspects of obesity could contribute to decidualization defects and that the number of human samples was small in this study, further research is required to underpin the molecular mechanisms that may lead to decidualization defects and, thus, poor receptivity in obese women.

Endometrial organoids can be derived and biobanked from fresh biopsies of infertile women who undergo IVF and monitored until a clinical pregnancy occurs. Organoids can also be established from frozen biopsies from infertile women, thus facilitating sample collection (Bui *et al.* 2020). By stratifying the patient samples according to maternal age, BMI and pregnancy outcome, specific comparisons to investigate organoid function could be done. A focus on organoids from women with BMI ≥ 30 or ≥ 40 years of age with implantation failure for whom dysfunctional endometrium might be a high-risk factor would be of great interest. Analysing the differences in their genetic signature or secretome profiles will allow identification of negative indicators of pregnancy success. Understanding how maternal age and bodyweight limit reproductive fitness will help clinicians offer specialized advice or even appropriate medical treatment to enhance the chances for a successful and healthy pregnancy.

The function of the female reproductive system is controlled by hormones released by the hypothalamus (GnRH), the anterior pituitary gland (FSH, LH, PRL) and the ovaries (oestrogen, progesterone). Endocrine-disrupting chemicals (pesticides, heavy metals, plasticizer alternatives, parabens) are commonly used in Western societies with concerns about their impact on female infertility (Rattan *et al.* 2017). REACH (Registration, Evaluation, Authorisation and Restriction of Chemicals), the European Union regulation which addresses the potential impact of chemical substances, has banished the use of di-(2-ethylhexyl) phthalate (DEHP), a plasticizer alternative, on grounds of causing toxicity. However, studies on how these chemicals disrupt female reproductive function are scarce. Organoids could be utilized to explore whether endocrine disruptors, highlighted by REACH as ‘high-concern substances’, cause endometrial toxicity using a reproductive toxicity reporter system. Recently, a dual reproductive organ-on-a-chip recapitulating the endocrine crosstalk between the ovary and the endometrium has been developed (Park *et al.* 2020). Although the study uses 2D cell cultures, this technology has great potential and incorporation of endometrial and ovarian organoid cultures would enable the accurate evaluation of the effect of various chemical substances on endocrine and, consequently, uterine function.

### Endometrium and its microbiome

Although the endometrium was always considered a ‘sterile’ environment, it is now accepted that commensals are present and pathogens can ascend through the cervix causing endometritis (Perez-Muñoz *et al.* 2017). Next-generation sequencing techniques revealed a microbiome signature (>90% *Lactobacillus* spp.) in the uterine mucosa that does not respond to hormonal changes and remains stable during the menstrual cycle (Moreno *et al.* 2016). It is possible that alterations in the microbiome resulting in inflammation could affect decidualization by activation of Toll-like receptors in epithelial or stromal cells (Benner *et al.* 2018). Furthermore, changes in the species (non-Lactobacillus dominated microbiota) in the receptive endometrium has been associated with a significant decrease in implantation and increased miscarriage in women who undergo IVF (Moreno *et al.* 2016), although the mechanisms responsible remain to be explored.

Colonization of the uterine tract with pathogenic bacteria or viruses leads to endometritis, a cause of infertility (Puente *et al.* 2020). Endometritis affects decidualization of human endometrial stromal cells by modifying the expression of ESRs and PGRs (Wu *et al.* 2017) but this study only had low sample numbers and needs further confirmation. 3D cultures of an endometrial epithelial cell line showed that infection with pathogenic *N. gonorrhoeae* induced proinflammatory mediators and morphological changes to the host cells (Łaniewski *et al.* 2017). We envisage that similar approaches could be employed with endometrial organoids to understand the mechanisms by which commensals and pathogens affect glandular integrity and differentiation and, ultimately, how this alters endometrial receptivity (Fig. 3). Indeed, murine endometrial organoids have been infected with *Chlamydia trachomatis* after being mechanically fragmented (Bishop *et al.* 2020), while more recently *Chlamydia* strains have directly been microinjected at the apical surface of the polarized epithelium (Dolat & Valdivia 2021). The infection resulted in reorganization of the cytoskeleton and the Golgi apparatus as well as disordered the cell junctions. Furthermore, the infected organoids were co-cultured with bone marrow-derived neutrophils, which could infiltrate the extracellular matrix (Matrigel) and migrate towards the infected organoids (Dolat & Valdivia 2021). Similar approaches could now be employed to infect endometrial organoids of human origin and test the efficacy of antimicrobial drugs in an effort to provide personalized therapy for women who suffer from endometritis.

## Endometrial organoids-on-a-chip

A major step forward in the generation of more complex *in vitro* models of reproductive tissues has been the application of microfluidic technologies. A ‘multi-organ’ microfluidic system that recapitulates aspects of the human menstrual cycle over 28 days by incorporating explants of the murine ovary, human fallopian tube, uterus, cervix and liver with steroid hormones released from ovarian follicles was developed (Xiao *et al.* 2017). Another approach is a dual reproductive organ-on-a-chip system that allows the bidirectional crosstalk between the ovaries and the endometrium. This model recapitulates the multicellular complexity of both tissues as the ovarian compartment contains granulosa and theca cells, whilst the endometrial compartment includes fibroblasts, vascular epithelial cells, immune cells, and endometrial stem cells (Park *et al.* 2020). Replacing tissue explants and 2D cell cultures with organoids would significantly enhance the accuracy of such multi-organ systems. They would be useful to investigate epithelial–stromal–immune cell interactions during different stages of the menstrual cycle and pregnancy and the effect of endocrine disruptors or pathogens and microbiota on reproductive function. Another exciting prospective in the context of obesity and diabetes would be a combination of organoids from the female reproductive tract (ovaries, endometrium) with those from adipose and pancreatic tissues to model systemic interactions and investigate how when perturbed, may affect fertility (Fig. 3).

## Translational applications of endometrial organoids for the treatment of infertility

### Personalized medicine

Despite numerous protocols for exogenous administration of oestrogen and progesterone to prepare the endometrium in women undergoing IVF, clinical pregnancy is not always guaranteed. There is considerable debate about the type, dosage and timing of hormonal supplementation (Nawroth & Ludwig 2005). Whilst oestrogen is crucial for the duration of the WOI, administration of high doses results in implantation failure in mice (Ma *et al.* 2003) and increases the risk of ovarian hyperstimulation syndrome (Aboulghar 2003). Likewise, the administration of progesterone in high concentrations for the treatment of luteal phase deficiency has a detrimental effect on uterine receptivity and decidualization, demonstrated in mice *in vivo* and immortalized human endometrial stromal cells *in vitro* (Liang *et al.* 2018).

Endometrial function varies considerably in women. Endometrial organoids offer a unique opportunity to provide personalized advice prior to hormonal treatment allowing the prevention of associated side effects like ovarian hyperstimulation syndrome. Organoids could be derived from endometrial biopsies of women undergoing IVF and exposed to different dose combinations of oestrogen and progesterone to evaluate proliferation and differentiation rates. Investigating the expression of ESR and PGR in the organoid cultures would also be useful for predicting the ability of the endometrium to acquire a receptive state in response to progesterone (Fig. 3).

Pharmacological therapies for endometriosis aim to suppress endogenous oestrogen production (aromatase inhibitors, gonadotropin-releasing hormone agonists/antagonists, selective oestrogen receptor modulators) and the activation of progesterone receptors (natural metabolites of progesterone, synthetic progestins, selective progesterone receptor modulators) (Rafique & Decherney 2017). Different patients may demonstrate distinct clinical symptoms, severity of disease or location of lesions that might affect the efficacy of the treatment. The latter should, therefore, be tailored for each patient using the recently established endometriotic organoids (Fig. 3). Thus, they could be a predictive tool for assessing the appropriate hormonal and pharmacological regimen to be administered to patients.

### Regenerative therapy

Endometrial regeneration after menstrual breakdown is unusual as it occurs without the fibrosis normally seen following ulceration of a mucosal surface. Scarring does develop if the basal layer is removed following uterine infection or surgery (dilation and curettage for pregnancy termination, cesarean section, myomectomy). The fibrous scarring can lead to intrauterine adhesions that are unresponsive to hormones and often asymptomatic. However, they can cause hypomenorrhoea/amenorrhoea, recurrent miscarriages and possibly infertility, which are all symptoms characteristic of Asherman Syndrome (AS). Attempts to lyse the fibrotic lesions are of questionable efficacy, with a high risk of recurrence (Bai *et al.* 2019). Cell-based therapies could provide an alternative for restoring endometrial tissue (Azizi *et al.* 2018). Human umbilical cord-derived mesenchymal stem cells on a collagen scaffold were transplanted into mice with artificially induced endometrial scarring to promote epithelial recovery and receptivity was enhanced (Xin *et al.* 2019). Autologous cells seeded on a biodegradable polymer scaffold could restore uterine structure and support pregnancies in rabbits (Magalhaes *et al.* 2020).

Organoids are an ideal source for regenerative medicine because they can be derived from very small tissue samples, expanded whilst retaining genomic stability and recapitulate epithelial tissue structure and cell types. Transplantation of autologous organoids derived from small endometrial biopsies to restore damaged epithelium would avoid allogeneic immune responses (Fig. 3). A similar approach to restore diseased or damaged organs has already been described using mouse colon and human liver organoids into mice (Yui *et al.* 2012, Huch *et al.* 2015). Critical issues that need to be addressed before this is adopted in the clinic are the safety of the graft, the timing of transplantation and the delivery process which needs to be targeted locally using biodegradable platforms. There is progress in the use of organoids for regenerative therapies as scalable GMP-grade human pancreas organoids for the treatment of Type 1 diabetes have been generated (Dossena *et al.* 2020). This is a promising step forward for devising similar approaches for the treatment of infertility and other endometrial disorders.

Organoids could also be used as an alternative therapeutic approach to treat progesterone-resistant lesions in endometriosis. Human-induced pluripotent stem cells differentiated to progesterone-sensitive endometrial stromal cells have been used to treat endometriosis (Miyazaki *et al.* 2018). Organoids could also be genetically manipulated to reinforce the expression of PGR and promote their response to progesterone and then be transferred back to the patient (Fig. 3). Reactivation of the PGR could also reinitiate the feedback loop that prevents continuous oestrogen-mediated proliferation. Although such therapeutic approaches require extensive optimization, they may increase the chances of fertility success in patients with endometriosis.

## Current limitations of endometrial organoid systems and future perspectives

Like any other model system, endometrial organoids have limitations. First, they can only be used to study epithelial cells and the absence of stromal and immune cells is a limitation for studying the interactions of all endometrial cell types. Efforts to generate a multicellular system or ‘artificial endometrium’ that integrates epithelial, stromal and immune cells using bioengineering approaches are needed. Such bioengineered tissues are potentially useful therapeutic approaches for cell replacement therapy in endometriosis and AS. A bioengineered endometrium will also provide an important tool to investigate the maternal–fetal interactions of early pregnancy using trophoblast organoids. Secondly, the apical surface of the endometrial organoids faces the lumen and therefore the development of more physiologically relevant models that expose the luminal surface will allow the study of implantation (Abbas *et al.* 2020). Thirdly, endometrial organoids are derived from patient samples leading to inevitable cellular heterogeneity and variability in behaviour and growth, similar to other organoid models (Lancaster & Huch 2019). Defining the normal levels of variability and increasing reproducibility are essential if organoids are to be used for personalized medicine and regenerative therapy (Lancaster & Huch 2019). The replacement of Matrigel, an animal-derived extracellular matrix, with fully synthetic matrices with amenable biochemical and biophysical properties that mimic the *in vivo* tissue architecture more closely will be an important step towards this goal (Hernandez-Gordillo *et al.* 2020). Further optimization of the composition of the media is also essential for organoid expansion or differentiation, and the use of microfluidics devices will lead to controllable and gradual administration of hormones and growth factors. The human element is also important and automation of organoid cultures could replace some of the variability introduced by different personnel handling the cultures with the added benefit of increasing scalability. Sensor systems could also be implemented for live assessment of the concentrations of the media components, hormones or other secreted products. Lastly, methods for derivation, culture and freezing of the organoids need to be fully compliant with good manufacturing practice. In summary, the endometrial organoid system in combination with emerging bioengineering technologies, gene editing and single-cell sequencing will transform the way we address and treat infertility of uterine origin to impact the lives of women.

## Conclusions

Infertility is a complex, multifactorial disorder and ~30% of cases still remain unexplained. The endometrium is the site of implantation and source of nutrition for the developing conceptus and its altered function is a major cause of infertility. However, because of the lack of physiologically relevant *in vitro* models of the human endometrium and the dynamic nature of this tissue, our understanding of normal endometrial function throughout the menstrual cycle remains limited. Although different conditions – physical anomalies (Asherman’s syndrome, thin endometrium), uterine disorders (endometriosis, adenomyosis, PCOS), altered immunological niche (microbiome imbalances, infections) and lifestyle factors (age, obesity) – affect fertility, understanding how these conditions affect endometrial epithelial behaviour is still limited. Generating organoids from healthy and pathological endometrium opens up opportunities to address these essential questions as well as promote personalized medicine approaches with tailored hormonal therapies and regenerative therapies for the treatment of infertility.

## Declaration of interest

The authors declare that there is no conflict of interest that could be perceived as prejudicing the impartiality of this review.

## Funding

K N was supported by the Wellcome Trust Investigator Award to A Moffett (UNS13724) and is now supported by a PhD fellowship from the Centre for Trophoblast Research. M Y T is supported by the Royal Society Dorothy Hodgkin Fellowship (DH160216) and has received funding from the European Research Council (ERC) under the European Union’s Horizon 2020 research and innovation programme (Grant agreement No. [853546]).

## Author contribution statement

K N and M Y T conceived and wrote the manuscript. K N prepared the figures.
